# Exercise intervention and improvement of negative emotions in children: a meta-analysis

**DOI:** 10.1186/s12887-023-04247-z

**Published:** 2023-08-22

**Authors:** Jiayu Li, Xiaoping Jiang, Zan Huang, Tianyi Shao

**Affiliations:** College of Physical Education and Health Sciences, Zhejiang Normal University, 321004 Jinhua, Zhejiang China

**Keywords:** Exercise, Children, Negative emotion, Meta-analysis

## Abstract

**Background:**

Anxiety, depression, and stress are the most common mental health problems in childhood. Exercise interventions in childhood help to promote mental health.

**Objective:**

To investigate the relationship between exercise interventions and improvement of negative emotions such as anxiety, depression, and stress in children (5–12 years).

**Methods:**

Articles were searched in five electronic databases from their inception to January 2023. The meta-analysis was performed using Stata 16.0.

**Results:**

Twenty-three intervention studies included 6830 children. 1) The exercise intervention group was significantly better than the control group in improving negative emotions (Standard Mean Difference SMD=-0.25, 95% Confidence Intervals CI: -0.34 to -0.15, P < 0.01). Exercise intervention improved different kinds of negative emotions: anxiety (SMD=-0.19, 95% CI: -0.33 to -0.06, P < 0.01), depression (SMD=-0.22, 95% CI: -0.43 to -0.01, P < 0.01), and stress (SMD=-0.33, 95% CI: -0.53 to -0.14, P < 0.01); it was most effective at relieving problematic stress. Exercise interventions lasting 20–45 min were most effective in improving children’s negative emotions (SMD=-0.38, 95% CI: -0.56 to -0.20, P < 0.01). An exercise intervention period of 10 weeks was more effective in improving children’s negative mood (SMD=-0.26, 95% CI: -0.34 to -0.17, P = 0.274).

**Conclusion:**

Exercise interventions may improve negative emotions such as anxiety, depression, and stress in children. These findings may have clinical implications for children with negative affect. However, these studies showed a large heterogeneity, and the results should be interpreted with caution. Future studies should report the variability of exercise interventions by gender, age group, and type, intensity, and place of exercise.

**Supplementary Information:**

The online version contains supplementary material available at 10.1186/s12887-023-04247-z.

## Introduction

Negative emotions reflect a general sense of distress and are often conceptualized as having different potencies, such as anxiety, depression, sadness, and anger [1]. Emotional problems, if left untreated, can continue to increase in severity, leading to poor mental health, disruption of social relationships, reduced academic performance, and a decline in overall quality of life [2, 3]. Since the start of the COVID-19 pandemic, 22.6% of children reported any depressive symptoms on the Children’s Depression Inventory-Short Form and 18.9% of children reported anxiety symptoms on the Screen for Child Anxiety Related Emotional Disorders [4]. Psychological problems caused by negative emotions often relapse, and the high incidence of depression and anxiety disorders caused by such psychological problems can even lead to premature death [5]. Absolutely, negative emotions have become a significant public health concern. One in eight people worldwide, suffer from emotional distress, with children aged 4–11 accounting for 1.57–6.9% of that number [6].

Childhood is a significant period for emotional development. Adverse emotional problems in childhood can affect emotion regulation during adolescence and adulthood [7]. The National Institutes of Health (NIH) defines the 5–12 years old population as children [8]. Children’s emotions are influenced by genetic, physiological, environmental, and life factors [9–12]. Although human beings can adjust their emotions, given that children are in a particular period of growth and development and that their brain organs are not yet fully functional, external interventions are necessary [13]. Educators and psychologists have alleviated children’s negative emotions through psychotherapy, cognitive therapy, and medication [14–16]. However, the widespread use of electronic devices and the increasing prevalence of sedentary behavior have reduced the effects of such interventions [17]. Consequently, there has been a notable increase in the amount of time children allocate to screen-based activities, such as engaging with social media, browsing the Internet, and participating in gaming, while simultaneously decreasing their involvement in offline endeavors such as face-to-face social interaction, sports/exercise, and attending religious services [18–20]. This alteration in habits has been accompanied by a notable rise in emotional difficulties among these children [21]. Depression typically necessitates a comprehensive and prolonged treatment strategy, entailing multiple financial considerations, including psychological consultations, medication, laboratory examinations, hospitalization, and other adjunctive treatment measures [22, 23]. The high cost of materials and time and space constraints make it challenging to implement treatment, and 1.6% of children with emotional problems are receiving treatment currently [24]. Nevertheless, this small percentage of children who receive treatment still need to achieve sustained gains. To improve children’s negative emotions, more researchers are looking at a tool for daily exercise [25–27].

In recent years, physical activities have been found to be beneficial to improve children’s negative emotions, whether spontaneous or organized [28, 29]. Sustained and appropriate length of physical exercise may alter the structure and function of the brain and can improve negative mood by increasing the brain’s concentrations of dopamine, serotonin, and norepinephrine [30]. Excessive and prolonged exercise may trigger the production of anabolic androgenic steroids, significantly increasing irritability and aggression and potentially triggering negative emotions [31]. Johnstone discovered that school sports activities can serve as a preventive measure against anxiety and depression among students [29]. Building upon this finding, Rodriguez-Ayllon expanded the research scope to explore the relationship between physical activity, sedentary behavior, mental illness, and mental health. However, their study did not specifically focus on the topic of “negative emotions” [32]. In a review by Song in 2021, it was revealed that both aerobic exercise and traditional Chinese exercise effectively alleviate depression symptoms in college students with an average age of 21.1 ± 1.55 years [33]. Wang demonstrated that a six-week duration of physical exercise can improve depression among teenagers aged 12–18 [34]. However, there is still a dearth of research concerning negative emotions in children. Considering the limitations of previous reviews and the emotional vulnerability of children, as well as the potential impact of exercise interventions on their emotional well-being, we selected “negative emotions” in children aged 5–12 as the study target and conducted a systematic review and meta-analysis of published English literature. Furthermore, this paper explored the effects of the exercise intervention duration, intervention period, and sample size on the study results.

## Materials and methods

### Protocol and registration

This review was performed according to Preferred Reporting Items for Systematic Reviews and Meta-Analysis (PRISMA) guidelines [35], and the Cochrane handbook for systematic reviews and meta-analysis [36]. The PRISSMA checklist is presented in Supplementary Material Table S1. This meta-analysis was registered in PROSPERO (number: CRD42022377772).

### Search strategy and information sources

A comprehensive search was done systematically through Scopus, PubMed, Web of Science, EBSCOhost, and APA PsycInfo up to the 30th of January 2023. Searching terms were based on adapted PICO questions to search through the aforementioned databases to accesses all the important articles. Free text words and medical subject heading (MeSH) terms were used. (1) (child* OR kid* OR enfant* OR toddler* OR pupil* OR “primary school student” OR boy* OR girl*); (2) (“physical activity” OR “physical exercise” OR “sports activities” OR “sport movement” OR sport* OR motor OR “athletic sports” OR “aerobic exercise " OR “aerobic training” OR “resistance exercise” OR “strength training” OR “muscle-strengthening exercise” OR “physical education” OR “fitness game”); (3) (anxiety OR anxious OR worry OR depression OR depressive OR depress* OR dumps OR pressure OR stress OR tension OR negative OR mood* OR affect* OR emotion* OR “psychological ill-being” OR “mental disease”). Details of the search strategy have been provided in Table S2. Finally, the references of the included studies and relevant studies were manually searched by senior experts in the field to supplement the electronic literature database search for missing literature. The search was performed independently by two researchers, and a third researcher was consulted in case of disagreement.

### Eligibility criteria of the selected studies

The inclusion criteria for the relevant studies were based on the PICOS (Participants/Interventions/Comparisons/Outcomes/Study Design) principles, as follows. Participants (P): relevant studies in children (5–12 years); Intervention (I): interventions in the form of exercise, such as aerobic exercise, fitness exercise, and physical education; Comparison (C): there was no artificially set physical exercise for the control group; Outcome (O): the primary outcome indicator of the study involved negative emotions in children; and Study Design (S): the study was intervention-based with a control group (including randomized controlled trials and non-randomized controlled trials).

The exclusion criteria for relevant studies were as follows: (1) non-English, unpublished literature, conference proceedings, theses, dissertations, and literature reviews; (2) studies of adults, animals, and special populations such as people with disabilities and psychiatric patients; (3) inability to extract valid data from the literature; (4) duplicate publications; and (5) inaccessible full text.

### Study selection

All references for the studies selected for this review were managed in EndNoteX9. After removing duplicates, the screening was conducted by separate researchers (XPJ, ZH). The researchers screened the remaining studies for eligibility by reviewing study titles and abstracts, then the full-text reports (JYL, XPJ) to evaluate their appropriateness to be included in the systematic review. For these steps, agreement was reached between two researchers and disagreement was resolved by consensus with a third researcher (TYS).

### Data extraction

Two researchers separately performed data extraction (ZH, XPJ). The extracted data were compared for all included studies, and disagreement was solved with a third reviewer (JYL). The extracted data included the first author’s name, year of publication, basic information about the study population (number, age range, country, and region), mood measures, type of intervention, and outcome indicators. The primary outcome indicator of the study involved negative emotions in children (Table S3).

### Assessment of quality of individual studies

This review assessed the included literature using the Physiotherapy Evidence Database (PEDro) scale, a credit rating scale developed by the Australian Centre for Evidence-Based Practice [37]. The scale consisted of randomized grouping (2 items), blinding (3 items), data reporting (3 items), data analysis (1 item), and follow-up (1 item), with a total of 10 criteria. Each item was recorded as 1 point when it appeared in the article and 0 points when it was not reflected, for a total score of 0 to 10 points. To avoid subjective opinions, two reviewers assessed the opinions, and the third judged the differences. The scale was scored out of 10, with scores ≥ 5 indicating high quality and scores < 5 indicating inferior quality.

### Summary measures

Meta-analysis was performed using Stata version 16.0. Effect sizes were statistically combined using standardized mean differences (SMDs) and 95% confidence intervals (CIs). Meta-regression was used to explore the extent to which covariates influenced inter-study heterogeneity. Subgroup analyses (i.e., intervention duration, intervention period, and type of negative affect) were conducted based on categorical variables, and used to determine which subgroup was more effective in improving negative affect. In addition, sensitivity analyses were conducted on the combined results to test the stability of the results [38].

### Synthesis of results

Each outcome was analyzed separately if the literature contained two or more negative emotion types. The meta-analysis was conducted by testing the heterogeneity of the included studies using *I*^*2*^, which quantifies the magnitude of heterogeneity and ranges from 0 to 100%; the more significant the *I*^*2*^ value, the greater the heterogeneity among the studies. If *I*^*2*^ ≤ 50%, the statistical heterogeneity among the studies was considered small, and the fixed-effects model was used for the meta-analysis; if *I*^*2*^ > 50%, there was significant statistical heterogeneity among the studies, and the randomized-effects model was used for the meta-analysis [39]. SMD sizes were interpreted using the guidelines provided by Cohen, where an effect size of 0.2 is considered a small effect, 0.5 is a medium effect, and 0.8 is a large effect [40].

### Publication bias

Funnel plots were drawn and Begg’s and Egger’s tests were used to detect whether the literature had publication bias [41].

## Results

### Literature screening process and results

**Fig. 1:**
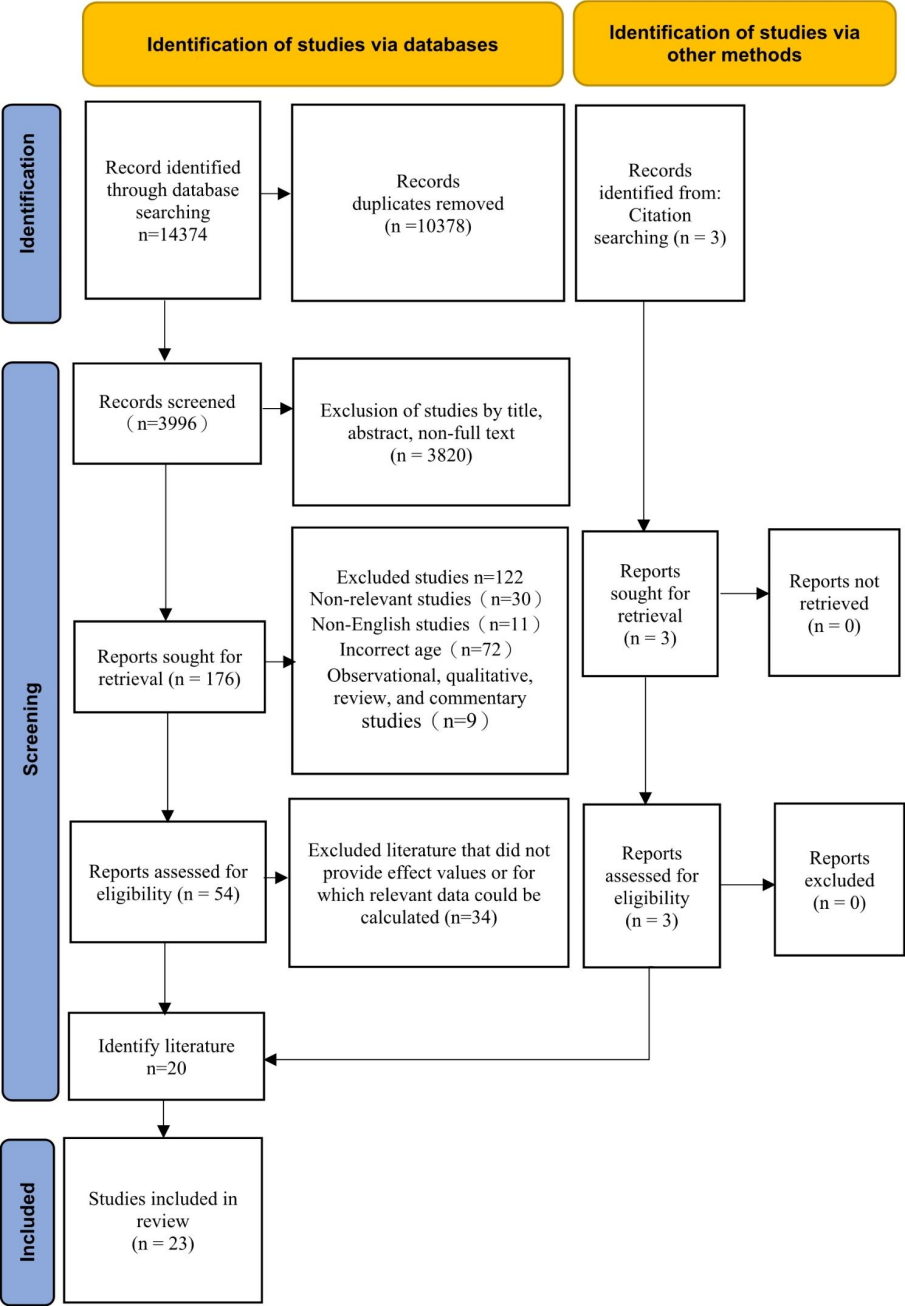
Flow chart of literature retrieval

After searching for subject terms, 14,374 pieces of literature were retrieved from the electronic database. First, the retrieved literature was imported into the literature management software Endnote and 3996 documents were obtained after removing duplicate documents. A total of 176 documents remained after reading the titles and abstracts and excluding non-full-text articles. Subsequently, the remaining documents were reviewed for full-text reading, and 54 full-text documents remained after excluding irrelevant documents. The effect values and 95% CIs or relevant data that could be calculated were verified and 20 articles remained. Three additional relevant articles were added through a manual search of the references in the included literature and relevant studies by senior experts in the field. A total of 23 articles were finally included in the meta-analysis of interventional studies. The specific search steps are shown in Fig. 1.

### Characteristics and evaluation of the quality of the literature

The basic characteristics of the included studies are presented in Table 1. The 23 studies included in the meta-analysis were published between 2001 and 2022, with 6830 respondents from 12 countries. Seven of the studies were based in the United States, five were based in Australia, two were based in Mexico; and one was based in Israel, Lithuania, Spain, Brazil, Germany, Sweden, India, Poland, and China. Eight studies had a sample size of fewer than 100 participants, accounting for 34.8% of the total included literature, and 15 studies had a sample size greater than 100 participants, accounting for 65.2% of the total included literature. In all studies, a variety of exercises were used in at least one trial group, and no exercise intervention or traditional curriculum was used in the control group. Measurements were recorded at each center by referencing or developing questionnaires and scales based on specific content to measure relevant indicators (Table S2). The subjects ranged in age from 5 to 12 years and were elementary school students (grades 2 to 6). By evaluating the quality of the included literature, 13 were found to be of high quality. (Table 2)

**Table 1:** Study Characteristics

Author (Year)	country	Sample Characteristics	Research Design	Sampling Type	Intervention Measure	Intervention Time	Testing Tool
Williamson [42]2001	The United States	64 children aged 9–10 years (34 boys, 30 girls)	Non-random allocation	Intervention research	Aerobic exercise: fun run, volleyball, space jump box, jump rope	15 min per session, two hours per week	The mood questionnaire
Crews [43] 2004	Spain	66 fourth grade children (33 girls, 33 boys)	Randomly assigned	Intervention research	Aerobic exercise	20 min each time, three times a week for 20 weeks	Trait Anxiety Inventory for Children and the Beck Depression Inventory
Shachar [44]2016	Israel	649 children in grades 3–6	Non-random allocation	Intervention research	Martial arts, soccer, basketball, volleyball, mini-soccer	20–45 min per session, 24 weeks	The 20-item Positive and Negative Affect Schedule
Romero-Perez [45]2020	Mexico	105 children, with an average age of 10.02 years	Randomly assigned	Intervention research	Aerobic exercise	50 min twice a week for 20 weeks	The Manifest Anxiety Scale in Children-Revised (CMAS-R) and the Depression Scale in Children (CDS)
Annesi [46]2005	The United States	26 girls and 23 boys with an average age of 10.5 years	Non-random allocation	Intervention research	Cardiovascular activities in the form of non-competitive tasks and games	30 min per session, 12 weeks	The Profile of Mood States-Short Form scales of Depression and Total Mood Disturbance
Farrell [47]2001	Australia	489 children aged 10–12 years, average age 10.54 years	Randomly assigned	Intervention research	Friends for Children(relaxation, cognitive restructuring, attentional training)	75 min per session for 10 weeks	The Spence Children’s Anxiety Scale (SCAS), The Revised Children’s Manifest Anxiety Scale (RCMAS)and The Children’s Depression Inventory (CDI)
Bazzano [48]2018	The United States	52 third graders	Randomly assigned	Intervention research	Small group yoga/positive thinking activities	40 min per session, 8 weeks	The Brief Multidimensional Students’ Life Satisfaction Scale-Peabody Treatment Progress Battery version (BMSLSS-PTPB)
Bohnert [49]2013	The United States	76 girls in grades 3–5, average age 9.13 years	Randomly assigned	Intervention research	Soccer, basketball, volleyball, tennis, golf, softball	90 min per session, 30 weeks	The Social Skills Scale of the Social Skills Rating System (SSRS)
Essau [50]2012	Germany	The average age of the 638 children aged 9–12 years was 10.91 years.	Non-random allocation	Intervention research	Friends program (including sports, sports games)	60 min per session, 10 weeks	The Spence Children’s Anxiety Scale (SCAS), Revised Child Anxiety and Depression Scale
Kall [51] 2015	Sweden	545 children in grades 4–6	Non-random allocation	Intervention research	“Games and sports” activities	30–45 min per session, two sessions per week	Strengths and Difficulties Questionnaire
Olive [52]2019	Australia	821 8-year-olds, 49% girls	Randomly assigned	Intervention research	Professional sports intervention programs (1) coordination and agility training, (2) skill activities, (3) motor challenges and games, (4) dynamic motor control, and (5) core movements.)	50 min each time, twice a week for four years	The Children’s Depression Inventory (CDI)and the Children’s Stress Questionnaire (CSQ)
Pophillat [53]2016	Australia	206 children aged 6–9, grades 1–3	Randomly assigned	Intervention research	Emotions and friends plan relaxation training, enjoyable activity schedule	10 weeks	The Children’s Depression Inventory (CDI), The Spence Children’s Anxiety Scale (SCAS)and The Assessment of Children’s Emotional Skills (ACES)
Roberts [54]2010	Australia	496 7th graders with an average age of 11.99 years	Randomly assigned	Intervention research	Australian Optimism Program (e.g., role play, games, and cooperative interactive activities)	60 min per session, 10 sessions for 20 weeks	The Child Depression Inventory (CDI), The Revised Children’s Manifest Anxiety Scale (RCMAS)and The Children’s Attributional Style Questionnaire – Revised (CASQ-R)
Rooney [55]2013	Australia	910 children, average age 8.75 years	Randomly assigned	Intervention research	Active Thinking Skills Program (AOPTP) Over Play and Physical Activity	60 min per session, 10 times per week for 30 months	The children’s depression inventory (CDI), Spence children’s anxiety scale (SCAS) and Strength and difficulties questionnaire (SDQ)
Telles [56]2013	India	98 school-age children, mean age 10.5 ± 1.3 years	Randomly assigned	Intervention research	Yoga, physical exercise including jogging, relay races or games	45 min of yoga or physical activity, five days a week for three months and 10 weeks	The Indian adaptation of Battle’s self-esteem Questionnaire
Wang [57]2022	China	366 children aged 5–6 years, (188 boys and 178 girls)	Non-random allocation	Intervention research	Gymnastics, squats, dance	40 min, 2 months, 5 weeks	Ages & Stages Questionnaires: Social-Emotional (ASQ:SE)
Weersing [58]2017	The United States	185 children, average age 11.3 (2.6) years	Randomly assigned	Intervention research	brief behavioral therapy (BBT)	45 min per session, 16 weeks	The Clinical Global Impressions scale
Wilczyńska [59]2022	Poland	57 children aged 9–12 years	Non-random allocation	Intervention research	Artistic gymnastics and rhythmic gymnastics, soccer	1 h per session, three times per week for 12 weeks	Sport Competition Anxiety Test (SCAT) and Competitive State Anxiety Inventory-2(CSAI-2RD)
Annesi [60]2004	Brazil	140 children from 7–11 (59 boys, 81 girls)	Non-random allocation	Intervention research	Gymnastics	40 min per class, three sessions	The Profile of Mood States Short Form scales of Tension and Depression
Andrade [61]2019	The United States	54 children from 9–12 (26 boys, 28 girls)	Non-random allocation	Intervention research	Various activities and non-competitive games	3 times a week for 12 weeks, 45 min at a time	Brunel Mood Scale
Cocca [62]2020	Mexico	252 children aged 10 to 12 years (133 boys, 119 girls)	Non-random allocation	Intervention research	Jumping, throwing, balancing, or catching the ball	Two 45-minute classes per week for 6 months	Beck Anxiety Inventory for youth; Stress in Children Questionnaire
Kliziene [63]2021	The United States	148 children aged 6–12 years	Randomly assigned	Intervention research	Running in place, dance moves and cartwheels	40–50 min each time 3 times a week for 8 weeks	The Revised Children’s Manifest Anxiety Scale
Gehricke [64]2022	Lithuania	364 children 6–9 (181 boys, 183 girls)	Randomly assigned	Intervention research	Basketball, soccer, gymnastics and track and field	45 min at a time, three days a week, for eight months	The screen for child anxiety related emotional disorders (SCARED)

**Table 2:** Quality Assessment of included studies

Author (Year)	Eligibility Criteria	Random Allocation	Concealed Allocation	Groups Similar at Baseline	Participants Blinded	Provider Blinded		Evaluator Blinded		Follow-Up	Intention-to-Treat Analysis	Between-Group Comparison		PEDro Score
Williamson [42] 2001	0	0	0	1	0		0	0	0		1	1	3	
Crews [43] 2004	0	1	0	0	0		0	0	0		1	1	3	
Shachar [44] 2016	1	0	0	1	0		0	0	0		1	1	4	
Romero-Perez [45] 2020	1	1	0	1	0		0	0	1		1	1	6	
Annesi [46] 2005	0	0	0	0	0		0	0	0		1	1	2	
Farrell [47] 2001	0	1	0	0	0		0	0	0		1	1	3	
Bazzano [48] 2018	1	1	0	1	0		0	0	0		1	1	5	
Bohnert [49] 2013	1	1	0	0	1		0	0	1		1	1	6	
Essau [50] 2012	0	1	0	0	0		0	1	1		1	1	5	
Kall [51] 2015	0	0	0	0	0		0	0	0		1	1	2	
Olive [52] 2019	0	1	0	1	0		1	0	1		1	1	6	
Pophillat [53] 2016	0	1	0	1	0		0	0	1		1	1	5	
Roberts [54] 2010	0	1	0	1	0		1	0	1		1	1	6	
Rooney [55] 2013	0	1	0	0	0		0	0	1		1	1	4	
Telles [56] 2013	1	1	0	1	0		0	1	1		1	1	7	
Wang [57] 2022	0	0	0	0	0		0	0	0		1	1	2	
Weersing [58] 2017	1	1	1	1	0		0	0	1		1	1	7	
Wilczyńska [59] 2022	1	0	0	1	1		0	0	0		1	1	5	
Annesi [60] 2004	0	0	0	0	0		0	0	0		1	1	2	
Andrade [61] 2019	1	0	0	0	1		0	0	0		1	1	4	
Cocca [62] 2020	1	0	1	0	1		0	0	0		1	1	5	
Kliziene [63] 2021	0	1	1	0	0		0	1	0		1	1	5	
Gehricke [64] 2022	1	1	0	1	0		0	0	1		1	1	6	

### Meta-analysis results

#### Exercise interventions help improve children’s negative emotions

**Fig. 2:**
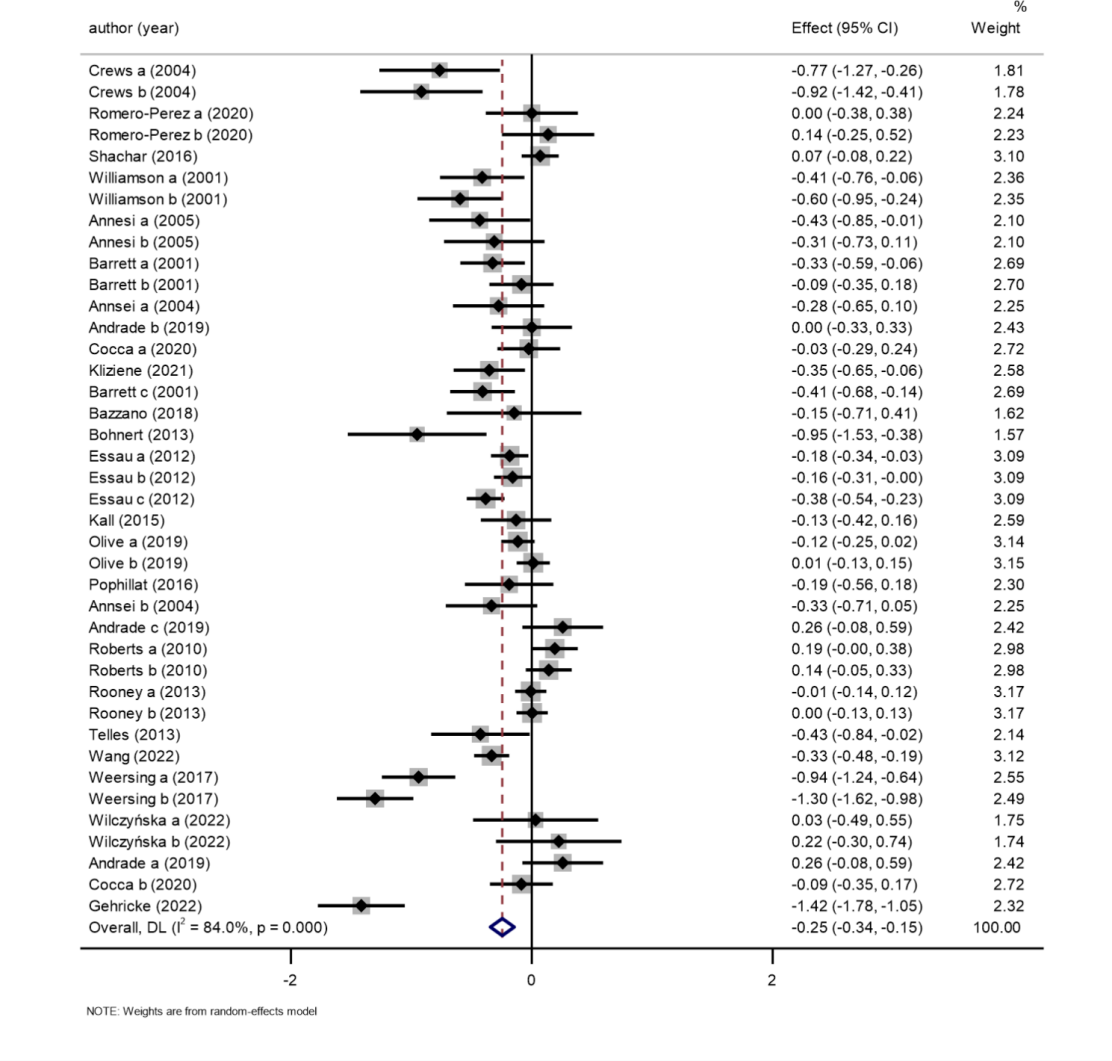
Forest plot of the association between the exercise and negative emotions in children

Twenty-three studies reported a relationship between children’s participation in physical exercise and changes in negative mood, using negative mood as an outcome indicator [42–64]. First, these 23 studies were tested for heterogeneity, and there was significant heterogeneity between studies (I^2^ = 84.0%>50%, P < 0.01). A random-effects model was selected to combine the results. The meta-analysis is shown in Fig. 2, which shows that the improvement in negative mood in the physical exercise participation group was significantly better than that in the control group (SMD=-0.25, 95% CI: -0.34 to -0.15, P < 0.01), indicating that participation in physical exercise could significantly reduce children’s negative emotions. Meta-regression was used to examine the source of heterogeneity in the combined results, with the combined effect value as the dependent variable and the year of publication of the literature, type of negative affect, duration of intervention, length of intervention, sample size, and geographic location of the country as meta-regression covariates to construct the model. The results of the univariate regression analysis are shown in Table 3, with P values greater than 0.05 for the year of publication (P = 0.242), type of negative affect (P = 0.386), duration of the intervention (P = 0.140), intervention period (P = 0.277), region (P = 0.517), and sample size (P = 0.121), indicating that these factors had no significant effect on all exercise interventions to improve negative affect.

**Table 3:** Meta-regression analysis of the effect of different factors on inter-study heterogeneity

Covariates	Regression Coefficient	Standard Error	T-value	P-value	95% CI	
					Lower Limit	Upper Limit
Year of publication	0.010	0.008	1.190	0.242	-0.007	0.028
Negative emotion type	-0.065	0.075	-0.880	0.386	-0.216	0.086
Intervention time	0.001	0.001	1.510	0.140	-0.001	0.003
Intervention period	0.057	0.052	1.10	0.277	-0.048	0.161
Region	0.041	0.062	0.650	0.517	-0.085	0.167
Sample size	-0.216	0.136	-1.580	0.121	-0.491	0.060

#### Subgroup analysis

To clarify the effects of different intervention characteristics (intervention duration and period) on negative emotions and their types, we conducted subgroup analyses on the types of negative emotions, intervention duration, and intervention period.

**Fig. 3:**
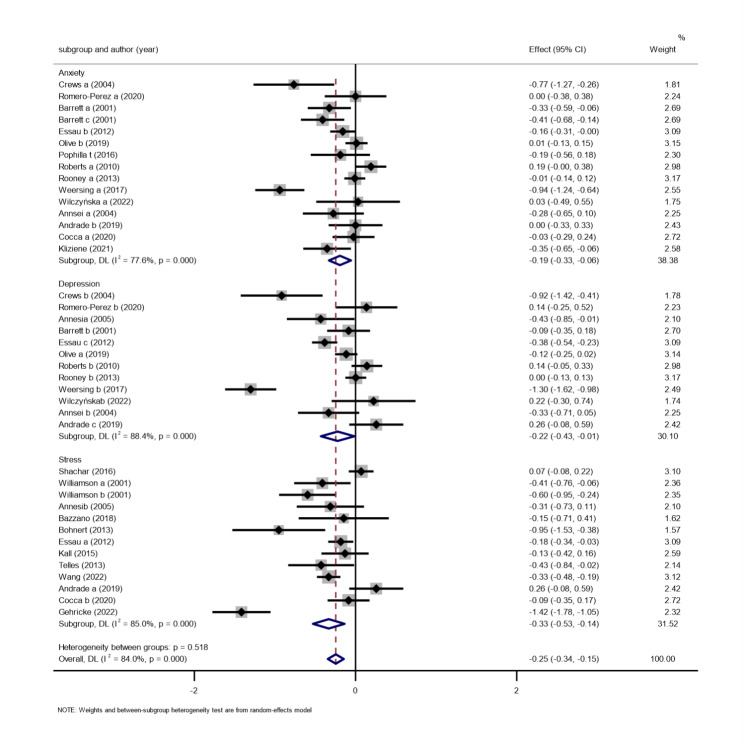
Subgroups of the effect of exercise intervention on different kinds of negative emotions

*Types of negative emotions.* A total of 23 studies, comprising 6830 participants, were included [42–64]. A meta-analysis using the random-effects model (Fig. 3) showed that exercise intervention had significant effects on improving different types of negative emotions in children. The exercise intervention groups performed better than the control groups. There were some differences in the total effect values in terms of improving different types of negative emotions in children. Fourteen studies showed a significant effect of exercise on improving anxiety, with a total effect value of (SMD=-0.19, 95%, CI: -0.33 to -0.06, P < 0.01). Eleven studies showed a significant effect of exercise on improving depression, with a total effect value of SMD=-0.22, 95% CI: -0.43 to -0.01, P < 0.01. The remaining 13 studies showed a significant effect of exercise on improving stress, with a total effect value of SMD=-0.33, 95% CI: -0.53 to -0.14, P < 0.01). This suggests that exercise interventions are the most effective in relieving stress in children.

**Fig. 4:**
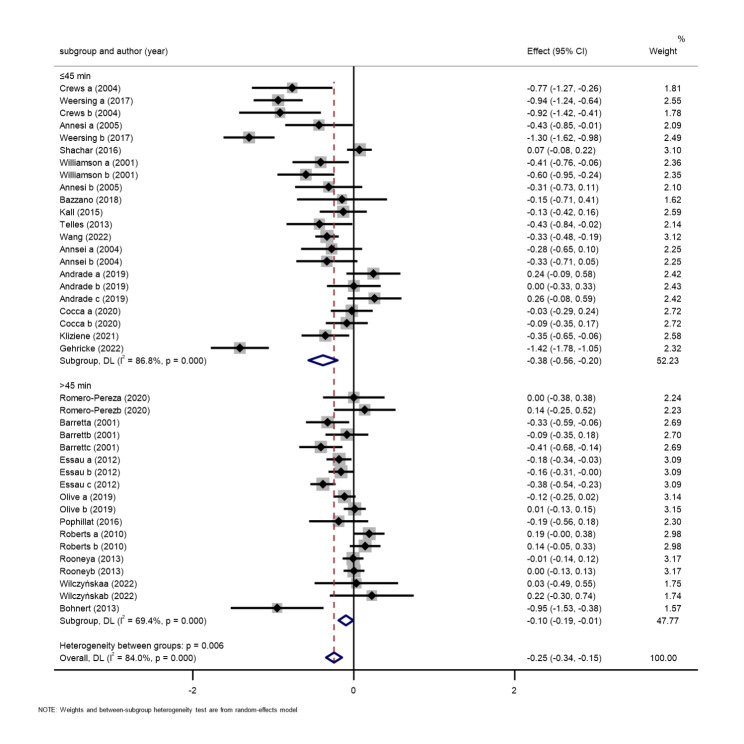
Exercise duration subgroup of the association between exercise and negative emotions in children

*Exercise intervention time.* A total of 23 studies, comprising 6830 participants, were included [42–64]. The results of the random-effects meta-analysis are shown in Fig. 4. The exercise intervention groups performed better than the control group. However, there were differences in the total effect values across intervention durations. The pooled effect value for 14 of the studies with an exercise duration ≤ 45 min was SMD=-0.38, 95% CI: -0.56 to -0.20, P < 0.01. The pooled effect value for nine studies with exercise duration more than 45 min was SMD=-0.10, 95% CI: -0.19 to -0.01, P < 0.01. The results showed that an exercise duration of 20–45 min was significantly more effective in improving children’s negative mood than an exercise duration over 45 min.

**Fig. 5:**
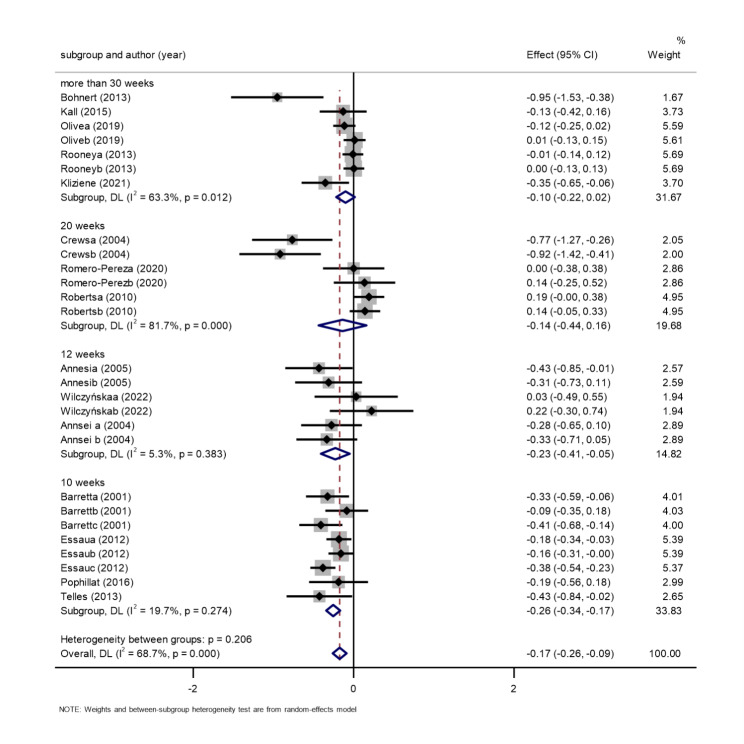
Intervention period subgroup of the association between exercise and negative emotions in children

*Exercise intervention duration*. Fifteen studies, comprising 4974 participants, were included [43, 45, 47–56, 59, 60, 63]. The results of the random-effects meta-analysis are shown in Fig. 5. The exercise intervention groups performed better than the control group. This suggests that all exercise interventions were effective in improving children’s negative emotions. However, there was some variation in the effects of different exercise intervention cycles on improving children’s negative mood. Four studies showed that the total effect value for the 10-week exercise intervention was SMD=-0.26, 95% CI: -0.34 to -0.17, P = 0.274. Three studies showed that the total effect value for the 12-week exercise intervention was SMD =-0.23, 95% CI: -0.41 to 0.05, P = 0.383. Three studies showed that the total effect value for the 20-week exercise intervention was SMD =-0.14, 95% CI: 0.44 to 0.16, P < 0.01. Five studies showed that the total effect value for exercise interventions longer than 30 weeks was SMD =-0.10, 95% CI: -0.22 to 0.02, P < 0.05. These results indicate that a 10-week exercise intervention was the most effective and effectiveness gradually decreased as the duration of the exercise intervention increased.

#### Sensitivity analysis

**Fig. 6:**
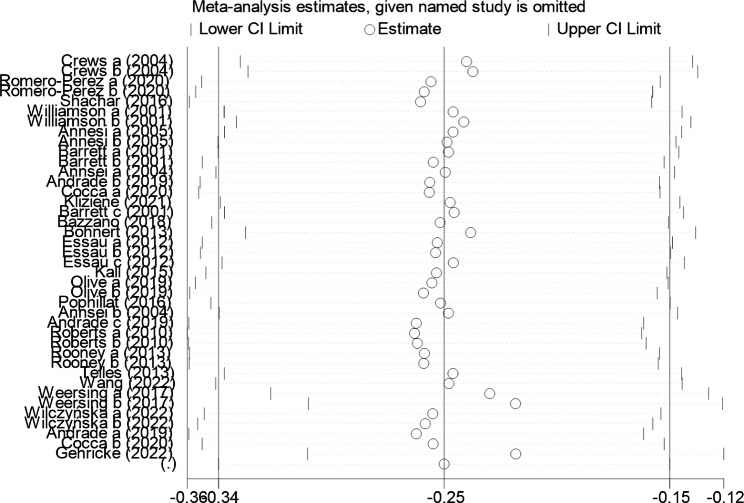
Sensitivity analysis of the association between exercise and positive emotions in children and adolescents

Sensitivity analysis was performed to further explore the sources of heterogeneity. Individual studies were excluded from analysis [42–64]. The results of the analysis are shown in Fig. 6. Consistent with the original analysis results, the individual studies had little effect on the combined results. This indicates that the combined effect values in this study were more stable.

#### Publication bias

**Fig. 7:**
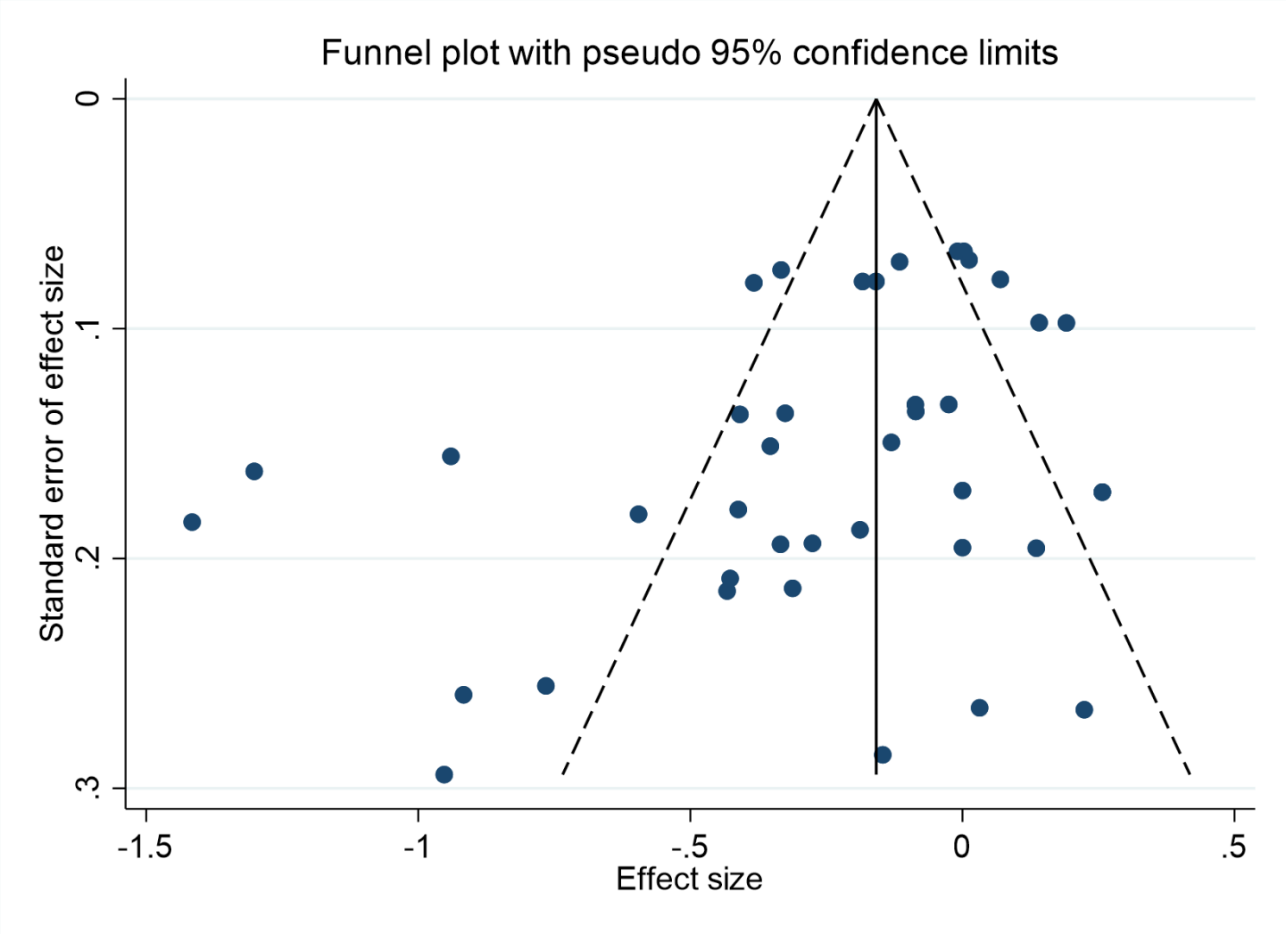
Funnel graph of publication bias of the association between exercise and positive emotions in children and adolescents

A publication bias test was performed on the included studies. As shown in Fig. 7, asymmetry is observed at the bottom right of the funnel plot. This predicted the possible existence of publication bias. However, this method is mainly based on subjective judgment, and may be inaccurate. Therefore, Begg’s and Egger’s tests were applied, and the results showed that P > 0.05. This indicates no significant publication bias in the literature.

## Discussion

This study presented a comprehensive quantitative analysis of the results of several independent studies on children’s physical exercise and negative emotions through a meta-analysis. A meta-analysis helped expand the total sample size and reduce selection bias in the study population. This compensates for the poor statistical efficacy and bias of an individual study and makes the conclusions more convincing [65]. The meta-analysis results showed a positive association between children’s participation in general physical exercise and improvement in negative mood, suggesting that children’s participation in physical exercise improves negative mood. This finding is similar to that of a review of the relationship between exercise and mental health conducted by Hale (2022) and others [66]. The 23 studies included in this meta-analysis were all intervention studies with a large sample size (6830), and each study had a more precise description of the selection of the study population; both the intervention and control groups were from the same population, and the study population was well represented. Meta-regression was used to test for sources of heterogeneity in the combined results. To clarify the effects of different intervention characteristics (intervention duration and period) on negative emotions and their types, we conducted subgroup analyses of intervention duration, intervention period, and negative emotion types. Participation in physical exercise significantly improved different types of negative emotions in children, such as anxiety, depression, and stress. The exercise intervention was the most effective in relieving stress in children’s negative emotions. An exercise duration of 20–45 min was the most effective compared to shorter and longer durations, and a 10-week exercise intervention period was more effective than shorter periods in improving children’s negative emotions.

### The relationship between exercise and improvement of children’s negative emotions

Given the current evidence, we can conclude that exercise interventions significantly improve negative moods (anxiety, depression, and stress) in children. However, the causal relationship between exercise and negative mood in children and its psychological and physiological mechanisms remain unclear, with most studies focusing on adolescents or adults. Nevertheless, four perspectives are currently available to help explain the improvement of negative mood in children by exercise: (1) the distraction perspective suggests that children are distracted from negative stimuli when participating in exercise, with significant improvements in mood both during and after the activity [67]. (2) The self-efficacy perspective suggests that exercise could be considered a challenging activity. Regular exercise may help increase self-confidence and counteract negative emotions [68]. (3) The social interaction perspective suggests that social relationships are inherent in physical exercise. Mutual support between individuals involved in physical exercise plays an important role in influencing positive emotions [69]. (4) The physiological view is that participation in exercise increases the synaptic transmission of monoamines and activates the secretion of endorphins [70]. These substances have inhibitory effects on the central nervous system, reducing pain and enhancing the active state of the brain. This results in mood improvement after exercise [71].

### The effect of exercise intervention on different kinds of negative emotions in children

Subgroup analyses showed that exercise significantly affected different types of negative emotions (anxiety, depression, and stress). First, in 2001, Sallis found that physical activity improved depression in children and adolescents through a review of 108 studies on physical activity [72]. A recent clinical medical study, which combines psychiatric and cardiological approaches and is highly persuasive, examined 210 patients with depression and found that increased depressive symptoms were associated with decreased physical activity [73]. In addition, some progress has been made in the study of exercise in improving anxiety. For example, Tao (2022) found that staying engaged in physical activity and reducing overly sedentary behavior further alleviated anxiety in a study investigating children with visual impairment [74]. This result is supported by a study by Gehricke [64]. In addition, we found that among the different types of negative emotions, motor intervention had the most significant effect on relieving stress in children’s negative emotions, with a practical value of -0.26. We speculate that stress is highly relevant to contemporary social development since most elementary school children are in a state of chronic academic stress [75]. Korczak et al. systematically evaluated the potential association between exercise and depression in children and adolescents. They found that this association was related to the type of exercise designed for the study [76]. In addition, anxiety, depression, and stress among negative emotions generally arise in two or more pairs; therefore, attention should be paid to the impact of multiple negative emotions that children may face in daily life.

### The effect of exercise intervention duration on children’s negative emotions

Subgroup analysis revealed that the length of the exercise intervention was a factor influencing negative mood. This study showed that performing about 20–45 min of exercise daily was most effective in improving children’s negative mood. The current clinical guidelines on the effect of exercise duration on children’s negative mood suggest that 45 min of moderate-intensity exercise at least three days per week improves negative mood [77]. This recommendation is consistent with the results of the present study. It has been shown that 20–40 min of aerobic exercise can improve anxiety in people with depression [78]. Another study retrospectively analyzed data from a 10-year longitudinal study and found that sustained exercise, even for durations as short as 15 min, was significantly associated with a reduced risk of depression [79]. In contrast, the Canadian government’s general health promotion guidelines and the American Academy of Pediatrics recommend that children and adolescents should engage in at least 60 min of moderate to vigorous exercise to maintain physical and mental health [80]. However, some studies have shown that prolonged exercise may trigger androgens in anabolic steroids [81]. The effects of steroids can cause significant increases in irritability and aggression, potentially triggering negative emotions.

### The effect of exercise intervention period on children’s negative emotions

Regarding the effect of the length of an exercise intervention cycle on children’s negative mood, the subgroup analysis of this study showed that exercise interventions for up to 10 weeks were the most effective. The extraordinary efficacy achieved with short-cycle exercise interventions is consistent with an earlier study on anxiety and depressive symptoms in children [82]. In a review of prevention analyses of anxiety in children and adolescents, Fisak found that the effects of interventions of 6 months in length and longer did not change significantly [83]. Our analysis of long-cycle interventions leading to lower effects may be due to (1) a natural decay process, (2) physical fatigue, (3) boredom with the environment, or a combination of all three [84, 85]. Further suggests that the effect of exercise intervention cycles does not increase with longer periods of time and that the effect of the intervention may decrease with increasing time. This also supports the optimal effect of the 10-week period mentioned in this study. Nevertheless, it is not possible to determine whether there are differences in the effects of exercise cycles on children’s negative affect. Due to the small number of included studies, which may affect the effect size statistics, further studies with large sample sizes are needed to verify the effectiveness of exercise intervention periods in the future.

## Limitations

This study has certain limitations. First, although a comprehensive and systematic search of the published literature was conducted, studies using other keywords may not have been included. Second, only English literature was included in this study, and relevant literature in other languages may have been excluded. Third, because most of the included studies reported aggregated data for each group only, the effect of underlying individual characteristics (e.g., age, gender, or baseline mood state) on the intervention effect was not considered, and aggregation bias may exist.

## Conclusion and outlook

This meta-analysis demonstrated that the effect of the exercise intervention on improving negative affect in children was significant, that is the exercise participation group was significantly better than the control group in improving negative affect. Exercise intervention significantly improved children’s negative emotions, such as anxiety, depression, and stress. It was most effective in relieving stress from children’s negative emotions. A 10-week exercise intervention with a controlled duration of 20–45 min was more effective in improving children’s negative emotions.

Based on the results of this review study, we hope that schools will actively encourage children to participate in exercise and control the duration of exercise for children to 20–45 min. When treating children with adverse mood-induced psychological problems, setting the exercise intervention period to 10 weeks may achieve better benefits, which has implications in clinical medicine.

This study highlights the need for further research on the effects of exercise interventions on improving children’s negative emotions. Future studies should explore the variations in exercise interventions across different genders, age groups, types of exercise, exercise intensity, and exercise settings, providing comprehensive and detailed insights. It is recommended to incorporate localized control variables and compare results with international findings to assess the psychological impact of exercise interventions on children in different countries. Additionally, the current study primarily relied on questionnaires or scales to measure relevant indicators, which may introduce bias. Therefore, future investigations could consider utilizing advanced emotion sensors to quantitatively assess children’s emotions, enhancing objectivity and validity in the research.

### Electronic supplementary material

Below is the link to the electronic supplementary material.

Supplementary Material 1

Supplementary Material 2

## Data Availability

All data generated or analysed during this study are included in this published article [and its supplementary information files].
