# Supplementary material for: Exercise intervention and improvement of negative emotions in children: a meta-analysis
Source: BMC Pediatr. 2023 Aug 22;23:411. doi: 10.1186/s12887-023-04247-z (PMC10464442; doi:10.1186/s12887-023-04247-z)
Supplement: Supplementary file 1 — Supplementary Material 1 [file 12887_2023_4247_MOESM1_ESM.docx]

**Table S2**, Search strategies

| Wos=3870 | ((TI=( child* OR kid* OR enfant* OR toddler* OR pupil* OR “primary school student” OR boy* OR girl*)) AND TI=(“physical activity” OR “physical exercise” OR “sports activities” OR “sport movement” OR sport* OR motor OR “athletic sports” OR “aerobic exercise” OR “aerobic training” OR “physical education” OR “fitness game”)) AND TS=(anxiety OR anxious OR worry OR depression OR depressive OR depress* OR dumps OR pressure OR stress OR tension OR negative OR mood* OR affect* OR emotion* OR “psychological ill-being” OR “mental disease”) |
| --- | --- |
| Scopus=4031 | ( TITLE ( child* OR kid* OR enfant* OR toddler* OR pupil* OR " primary school student " OR boy* OR girl* ) AND TITLE ( "physical activity" OR "physical exercise" OR "sports activities" OR "sport movement" OR sport* OR motor OR "athletic sports" OR "aerobic exercise" OR "aerobic training" OR "physical education" OR "fitness game" ) AND TITLE-ABS-KEY ( anxiety OR anxious OR worry OR depression OR depressive OR depress* OR dumps OR pressure OR stress OR tension OR negative OR mood* OR affect* OR emotion* OR "psychological ill-being" OR "mental disease" ) ) |
| Pub med=2452 | ((child*[Title] OR kid* [Title] OR enfant*[Title] OR toddler*[Title] OR Pupil*[Title] OR primary school student[Title] OR boy*[Title] OR girl*[Title]) AND ("physical activity"[Title] OR "physical exercise"[Title] OR "sports activities"[Title] OR "sport movement"[Title] OR sport*[Title] OR motor[Title] OR "athletic sports"[Title] OR "aerobic exercise"[Title] OR "aerobic training"[Title] OR "physical education"[Title] OR "fitness game"[Title])) AND (anxiety[Title/Abstract] OR anxious[Title/Abstract] OR worry[Title/Abstract] OR depression[Title/Abstract] OR depressive[Title/Abstract] OR depress*[Title/Abstract] OR dumps[Title/Abstract] OR pressure[Title/Abstract] OR stress[Title/Abstract] OR tension[Title/Abstract] OR negative[Title/Abstract] OR mood*[Title/Abstract] OR affect*[Title/Abstract] OR emotion*[Title/Abstract] OR "psychological ill-being"[Title/Abstract] OR "mental disease"[Title/Abstract]) |
| APA PsycInfo=1458 | TI ( child* OR kid* OR enfant* OR toddler* OR Pupil* OR primary school student OR boy* OR girl* ) AND TI ( “physical activity” OR “physical exercise” OR “sports activities” OR “sport movement” OR sport* OR motor OR “athletic sports” OR “aerobic exercise” OR “aerobic training” OR “physical education” OR “fitness game” ) AND AB (anxiety OR anxious OR worry OR depression OR depressive OR depress* OR dumps OR pressure OR stress OR tension OR negative OR mood* OR affect* OR emotion* OR “psychological ill-being” OR “mental disease”) |
| EBSCOhost ERIC=2563 | AB ( child* OR kid* OR enfant* OR toddler* OR Pupil* OR primary school student OR boy* OR girl* ) AND AB ( “physical activity” OR “physical exercise” OR “sports activities” OR “sport movement” OR sport* OR motor OR “athletic sports” OR “aerobic exercise” OR “aerobic training” OR “physical education” OR “fitness game” ) AND AB (anxiety OR anxious OR worry OR depression OR depressive OR depress* OR dumps OR pressure OR stress OR tension OR negative OR mood* OR affect* OR emotion* OR “psychological ill-being” OR “mental disease” ) |

**Table S3**: Tool Description

| Author / Year | Tool | Tool Description |
| --- | --- | --- |
| Williamson 2001 | The mood questionnaire | a self-report mood measure of eight positive and eight negative adjectives |
| Crews 2004 | Trait Anxiety Inventory for Children and the Beck Depression Inventory | Trait Anxiety Inventory for Children is used to evaluate trait anxiety, while the Beck Depression Inventory is used to evaluate depression. |
| Shachar 2016 | The 20-item Positive and Negative Affect Schedule | a self-report checklist of affect adjectives, was designed to provide independent measures of positive and negative affect. |
| Romero-Perez 2020 | The Manifest Anxiety Scale in Children-Revised (CMAS-R) and the Depression Scale in Children (CDS) | CMAS-R is a self-administered questionnaire, aimed at children and adolescents between 6 and 18 years old, composed of 37 items and four subscales: physiological anxiety, restlessness, social concerns, and lies. The CDS consists of a self-report questionnaire, directed at children between 8 and 16 years old, composed of 66 statements, 48 of them of a depressive type and 18 of a positive type. |
| Annesi 2005 | The Profile of Mood States-Short Form scales of Depression (5 items) and Total Mood Disturbance (30 items) | to assess depression and overall negative mood. Total Mood Disturbance is an aggregate assessment of mood which is derived by summing the Profile of Mood States ratings on Tension, Fatigue, Depression, Confusion, and Anger, then subtracting Vigor. |
| Barrett 2001 | The Spence Children's Anxiety Scale (SCAS), The Revised Children's Manifest Anxiety Scale (RCMAS)and The Children's Depression Inventory (CDI) | SCAS is a self-report measure of anxiety designed for use with children aged 8–12 years. RCMAS consists of 28 items assessing a child’s chronic or trait anxiety and 9 items assessing social desirability. CDI has 27 items related to the cognitive, affective, and behavioral signs of depression. |
| Bazzano 2018 | The Brief Multidimensional Students' Life Satisfaction Scale-Peabody Treatment Progress Battery version (BMSLSS-PTPB) | BMSLSS-PTPB was used to assess student satisfaction at baseline, midline, and endline surveys. |
| Bohnert 2013 | The Social Skills Scale of the Social Skills Rating System (SSRS) | The self-report version of the SSRS Social Skills Scale consists of 4 subscales including Cooperation, Assertion, Empathy, and Self-Control. |
| Essau 2012 | The Spence Children's Anxiety Scale (SCAS), Revised Child Anxiety and Depression Scale | SCAS is a 38-item measure of anxiety symptoms in children and adolescents. RCADS consists of11 items that correspond to the nine symptoms of Criterion A for major depressive episode. |
| Kall 2015 | Strengths and Difficulties Questionnaire | SDQ consists of 5 scales, each consisting of 5 items. The 5 scales measure difficulties in 5 psychological domains: emotional symptoms, conduct problems, hyperactive-inattention, peer relationship, and prosocial behavior. |
| Olive 2019 | The Children's Depression Inventory (CDI)and the Children's Stress Questionnaire (CSQ) | The CDI is mainly used to measure depression-related symptoms. The CSQ is a 50-item inventory assessing stressor exposure and the impact of self-reported stressor experience over the past 12 months. |
| Pophillat 2016 | The Children’s Depression Inventory (CDI), The Spence Children’s Anxiety Scale (SCAS)and The Assessment of Children’s Emotional Skills (ACES) | CDI is a 27-item self-report measure that assesses cognitive, affective and behavioral symptoms of depression in children aged 7–17. SCAS is a child self-report measure of symptoms relating to six subsets of anxiety disorders: social phobia, separation anxiety, obsessive-compulsive disorder, panic-agoraphobia, generalized anxiety and fears of physical injury in children aged 8 and above. ACES was used to assess children’s emotional knowledge. |
| Roberts 2010 | The Child Depression Inventory (CDI), The Revised Children’s Manifest Anxiety Scale (RCMAS)and The Children’s Attributional Style Questionnaire – Revised (CASQ-R) | CDI assessed depressed affect, somatic symptoms, depressive behavior, low self-esteem, and anhedonia. RCMAS assessed physiological symptoms, worry, over-sensitivity, social and concentration concerns. CASQ-R assessed attribution style for positive and negative events in 8–13-year-olds. |
| Rooney 2013 | The children’s depression inventory (CDI), Spence children’s anxiety scale (SCAS) and Strength and difficulties questionnaire (SDQ) | The CDI is a 27-item, self-rated symptom-oriented instrument for assessing depression in children between the ages of 7–17 years. The SCAS was developed to assess the severity of anxiety symptoms in line with anxiety disorders proposed by the DSM-IV. SDQ is a 25-item behavioral screening questionnaire measuring parental perceptions of internalizing and externalizing problems for children and adolescents aged 4 through to 16 years. |
| Telles 2013 | The Indian adaptation of Battle’s self-esteem questionnaire | The questionnaire has 50 close-ended questions with 4 subscales. |
| Wang 2022 | Ages & Stages Questionnaires: Social-Emotional (ASQ:SE) | The questionnaire contains 33 questions, each of which characterizes a specific aspect of a child’ s behavior: self-regulation, compliance, communication, adaptive behaviors, autonomy, affect, and interaction with people. |
| Weersing 2017 | The Clinical Global Impressions scale | The Clinical Global Impressions scale was used to assess global severity (CGI-S) and improvement (CGI-I) across anxiety and depression. |
| Wilczyńska 2022 | Sport Competition Anxiety Test (SCAT) and Competitive State Anxiety Inventory-2(CSAI-2RD) | The CSAI-2RD consists of a 14-item scale containing cognitive anxiety statements and somatic anxiety statements. |
| Annsei 2004 | the Profile of Mood States Short Form scales of Tension and Depression | Individuals rated how much specific psychological states were felt over the past week with anchors of O =Not at all and 4 =Extremely. |
| Andrade 2019 | Brunel Mood Scale | BRUMS evaluates six mood dimensions: three psychological states (feeling of depression, anger, and mental confusion) and three psychosomatic states (fatigue, tension, and vigor). |
| Cocca 2020 | Beck Anxiety Inventory for youth; Stress in Children Questionnaire | Beck Anxiety Inventory for youth, consisting of 20 items with answers ranging from 0 (never) to 3(always). Stress in Children Questionnaire contains 16 items with Likert Scale from 0 (none) to 3 (always). Higher total scores (sum of items’ answers) indicate higher level of stress. |
| Kliziene 2021 | The Revised Children’s Manifest Anxiety Scale | The Revised Children’s Manifest Anxiety Scale (RCMAS) contains 37 items with 28 items used to measure anxiety and an additional 9 items that present an index of the child’s level of defensiveness. |
| Gehricke 2022 | The screen for child anxiety related emotional disorders (SCARED) | The SCARED is a child self-report instrument used to screen for childhood anxiety disorders including general anxiety disorder, separation anxiety disorder, panic disorder and social phobias. |
